# The Association Between Community Stressors and Asthma Prevalence of School Children in Winnipeg, Canada

**DOI:** 10.3390/ijerph9020579

**Published:** 2012-02-16

**Authors:** Tyler P. Pittman, Candace I. J. Nykiforuk, Javier Mignone, Piush J. Mandhane, Allan B. Becker, Anita L. Kozyrskyj

**Affiliations:** 1 Department of Pediatrics, University of Alberta, Edmonton, AB T6G 2J3, Canada; Email: kozyrsky@ualberta.ca; 2 Cancer Surveillance, Surveillance and Health Status Assessment, Population and Public Health, Alberta Health Services, Edmonton, AB T5J 3H1, Canada; 3 Centre for Health Promotion Studies, University of Alberta, Edmonton, AB T6G 1C9, Canada; Email: candace.nykiforuk@ualberta.ca; 4 Department of Family Social Sciences, University of Manitoba, Winnipeg, MB R3T 2N2, Canada; Email: mignonej@cc.umanitoba.ca; 5 Division of Respiratory Medicine, Department of Pediatrics, University of Alberta, Edmonton, AB T6G 2J3, Canada; Email: mandhane@ualberta.ca; 6 Department of Pediatrics & Child Health, University of Manitoba, Winnipeg, MB R3A 1S1, Canada; Email: becker@cc.umanitoba.ca; 7 Women and Children’s Health Research Institute/Stollery Children’s Hospital Foundation, Edmonton, AB T6G 2C8, Canada; 8 School of Public Health, University of Alberta, Edmonton, AB T6G 1C9, Canada; 9 Department of Community Health Sciences, University of Manitoba, Winnipeg, MB R3E 0W3, Canada

**Keywords:** childhood asthma, community stressors, multilevel modelling

## Abstract

It is generally surmised that community stressors have an incubating effect for a variety of diagnoses on maternal and child health. This is of public health significance, as children of mothers facing long-term distress were found to have a 60% higher risk for asthma diagnosis at age 7 in Manitoba, Canada. Our objective was to determine the association of community stressors with childhood asthma prevalence in Winnipeg, Canada from participants who completed the Study of Asthma, Genes and the Environment (SAGE) survey administered in 2002–2003 to a birth cohort from 1995. Measures of *community socioeconomic makeup* and *community disorder* with rank ordinalized by quintile at the census tract level were obtained from the 1996 Canada Census. Crime data (annual incidence per 10,000 persons) by neighbourhood profile for 2001 was provided by the Winnipeg Police Service. Dichotomous caregiver report of child asthma along with other indicators from the geocoded SAGE survey allowed linkage to 23 neighbourhood profiles. Multilevel logistic regression analyses were performed to estimate the effect of community stressors on childhood asthma prevalence for birth and non-birth home children (*N* = 1472) and children resident of birth homes at age 7 or 8 (*N* = 698). After adjusting for individual risk factors, children resident of birth homes in a high thefts over $5,000 neighbourhood profile were twice as likely (Adjusted OR, 2.05; 95% CI, 1.11–3.81) to have report of asthma compared to children in a lower thefts over $5,000 profile, with community thefts over $5,000 explaining over half of the observed neighbourhood variation in asthma.

## 1. Introduction

Hospitalization rates for asthma disproportionately burden economically disadvantaged youth residing in urban communities [1]. Increasingly, asthma prevalence amongst children of low income and minority populations exceeds that of the aggregate population or higher income groups [2,3,4,5]. A sustained increase of asthma incidence rates among school children have been observed globally since the 1970s, with the hypothesized link to air pollution and other environmental factors remaining unproven [6]. This comes at a time when there is recognition that even the most expensive and complex of medical procedures are unable to dissipate levels of childhood chronic disease [7]. It is increasingly evident that in addition to poverty, the chronic stress of living in a neighbourhood affects the health of children. Community stressors influence not only the health of the individual, but reduce the capacity of the population in a neighbourhood to withstand strain [8,9,10,11,12]. As a morbidity with multifactorial pathways for disease onset, asthma has been associated with a variety of lifestyle practices, genetic expression and environmental exposure specific to each person [13]. However, genetic background alone is unlikely to account for short-term changes in the rise and prevalence of asthma cases, with lifestyle and environment being more plausible influences [14,15]. Numerous articles have been published in recent years investigating the impact of the social and physical environment on adult or adolescent asthma populations, with few studies targeting asthma prevalence among school children in the Canadian context [16,17,18].

The socioeconomic makeup of a neighbourhood as characterized by household income, level of employment and extent of home ownership, has been shown to contribute substantially to mental well-being of its adult female members [11,19,20,21]. In addition to positively impacting caregiver mental health, neighbourhoods with higher affluence or less economic disadvantage also promote child health through for example, greater social exchange on child development [22]. On the other hand, neighbourhood social disorder is less likely to promote personal interaction or opportunities for social activity [20]. Social disorder itself, seen as a landscape encompassing poorly maintained buildings and streets with high rates of litter, vandalism, and noise (from traffic and other homes) can be a direct antecedent to stress and distress among mothers [8,12]. At the other extreme, community crime denotes a breakdown in traditional social structures, and facilitates an environment of fear, as well as psychosocial distress. New hypotheses have been put forward for the toxic role of high violence neighbourhoods in promoting high asthma prevalence rates in inner-city U.S. neighbourhoods [23]. More recent multilevel analyses on community violence and childhood asthma amongst African-American youth in Chicago showed that children residing in high crime neighbourhoods had a 56% higher likelihood of asthma than those in low crime neighbourhoods, after adjusting for individual-level covariates such as maternal asthma and family violence in home [24]. 

Also, there is increased awareness of the linkage between neighbourhood safety and resources available to mothers in the pre- and postnatal period and their stress levels. Distress among pregnant women has been reported to be clustered in predominantly low income neighbourhoods, characterized by measures of incivility that include social interactions, housing conditions and litter in public spaces [20]. The contribution of external pressures and stress has been described as a “double jeopardy” situation for maternal health and mental well-being, in that negative exposures influencing poor health of mothers often translate to an environment of less optimal outcome for children [25]. A community environment high in psychosocial stressors can foster a setting of anxiety and depression in mothers and children [26]. Children of mothers with depressive symptoms have been shown to have more behavioral problems and poorer health, which in turn exacerbates the distress of the mother [27]. A Canadian population-based birth cohort study found children to be at a 25% increased risk for asthma by age 7 if their mothers faced long-term distress, as measured by health care or prescription medication utilization for anxiety or depression [28]. 

Stress related to the psychosocial environment is increasingly recognized as an equally important risk factor for childhood asthma as indoor and outdoor air [6]. The evidence for the most part, indicates that low household socioeconomic status contributes to asthma development in children [5,29,30], although the reverse association has been observed with ecologic-based measures of socioeconomic status [15,31]. Furthermore, aside from socioeconomic indicators, little information exists on the effects that community stress, characterized under the domains of *social disorder*, and *crime and violence* may have on the risk of asthma in Canadian schoolchildren, independent of household level exposures. The goal of the present study is to explore the association between community stressors and parent report of childhood asthma, and the contribution of these stressors to socioeconomic inequalities in asthma prevalence. Our study in particular examines the association of community crime on asthma prevalence of children in Winnipeg neighbourhoods, after adjusting for household exposures. 

## 2. Experimental Section

### 2.1. SAGE Survey in Manitoba

This was an ecologic analysis of data from the Study of Asthma, Genes and the Environment (SAGE) survey administered in 2002–2003. Consent and ethics approval were obtained from the Health Research Ethics Boards at the University of Manitoba and the University of Alberta. SAGE is a longitudinal study of a complete birth cohort for the year 1995 in the province of Manitoba, derived from the provincial health care registry to study early life environmental exposures and the development of asthma in childhood. Full details of the SAGE study have been published elsewhere [29]. Out of 12,556 questionnaires mailed out through Manitoba Health to caregivers of children in 2002–2003, 3598 were returned representing a response rate of 28.6%. Although this is a modest response rate, it is in keeping with population-wide mail surveys derived from health registries [29]. Further, the urban-rural distribution of child asthma was similar to provincial statistics. Survivor sampling was employed, in that controls were selected at end of follow-up (survey return) as subjects with no parent report of child asthma. Cases were defined as children with caregiver report of asthma. An indicator denoting if child was resident of birth home at age 7 or 8 was available from respondents in the City of Winnipeg and used in sensitivity analyses to test the effect of community stressors from living in the same neighbourhood since birth. Approximately half of the children (52.6%) inhabited a different home at age 7 or 8. 

Prevalence of child asthma by caregiver report was approximately 12% for the province of Manitoba among 3596 participants in the SAGE survey. This compares to national asthma prevalence rates of 13% in Canadian children [32]. Among respondents of birth homes in the City of Winnipeg, the percentage of cases of parent-declared child asthma was also 12% (84/698) from the SAGE survey. Conversely, amongst birth and non-birth home children age 7 or 8 in Winnipeg the asthma percentage was 13.4% (197/1472). These findings are in agreement with the asthma prevalence rate of 10% in southern rural areas and 14% in urban areas for Manitoba respectively [33]. It has been suggested that population-based cross-sectional surveys do not reflect the true prevalence of asthma (because of under-estimation) since they are dependent upon parent report of symptoms and not physician diagnosis [6]. 

Household measures of interest from the SAGE survey that were examined in detail, based on their inclusion in other studies, included: mother, father and family history of asthma, hay fever in child, mold in household 1995, and smoking in household [15,24,29,31,34]. Minimal data clean-up on the SAGE survey was necessary to exclude respondents that could not be geocoded through use of the Postal Code Conversion File (PCCF) [35]. Two records were removed, one that listed a non-Canadian postal code, and another which provided no postal code.

### 2.2. Census Tract and Neighbourhood Profile Selection Criteria

Grouping units were defined at two levels: (i) census tracts (*N* = 133); and (ii) City of Winnipeg neighbourhood profiles (*N* = 23). Census track boundaries for the City of Winnipeg were nested within neighbourhood profiles, with some divergence of geographies noted in the predominantly industrial areas for the profiles of Fort Garry South, Seven Oaks West and River Heights West [36]. Census tracts with census tract unit identifier (CTUID) codes from the 1996 Canada Census demarcating units outside the City of Winnipeg municipal boundaries, or for those which there were no study respondents were excluded [37]. The Postal Code Conversion File (PCCF) version 5D was used to assign latitude and longitude coordinates to the postal codes of residence provided by respondents of the SAGE survey, in addition to CTUIDs [35]. Roughly 40.9% (1472/3596) of SAGE survey children resided in the census tracks and neighbourhood profiles comprising Winnipeg. The median number of children in each census tract was 9 (IQR, 5–15), and 64 (IQR, 33–91) per Winnipeg neighbourhood profile.

### 2.3. Measures of Community Socioeconomic Makeup and Community Disorder

Environmental profiles were created using data from the time period closest to 1995, the birth of SAGE children. This was done to ensure that exposure to community stressors preceded onset of asthma as much as possible. Stressors characterized under *community socioeconomic makeup* and *community disorder* domains were extracted from the 1996 Canada Census [37], as these measures provided context to the effect of neighbourhood in the pre- and postnatal time period. In addition, follow-up analyses showed that residential location of asthma respondents from the SAGE survey had higher correlations with SES indicators from the 1996 Canada Census compared to those from the 2001 or 2006 censuses. 

To be able to compare scores to other SES indicators gathered on an ordinal scale, and ease computation for the multilevel logistic models, a relative ranking score from one to five (quintile) was assigned to each CTUID denoting a stressor value from 1996 Census in reference to all other census tracts within the Winnipeg CMA. Principal component analysis (PCA) was used to guide the separation of indicators provided from the census into two roughly orthogonal domains, but were not tested in models as a newly-created PCA group as proposed by other studies [38,39,40]. The grouping for measures encompassing the first hypothesized domain of *community socioeconomic makeup* included indicators related to census tract proportion of: Aboriginal, aged 15+ and unemployed, average household income, female lone parent household, home owner household, income government transfer comprise, lone parent household, low income, males aged 15+ and unemployed, median household income, moved last 5 years, no school diploma and aged 15+, owner housing unaffordable, population change last 5 years, population density, population married, ratio non-workers to workers, recent immigrant, rental household, renter housing unaffordable, standard error average income and visible minority. The grouping for measures of the second hypothesized domain, *community disorder* consisted of proportion of children aged 5 to 14, homes needing major repairs, labour force participation, males aged 15 to 24, households with no income, senior population, as well as average family size.

### 2.4. Measures of Community Crime

The year 2001 was chosen for capture period with crime statistics, as it corresponded to a census year and preceded mail out of the SAGE survey in Manitoba. It is also one of the earliest periods that Winnipeg Police Service has community crime data aggregated at the neighbourhood-level available. The population of the aggregated census tracts from the 2001 Canada Census nested within City of Winnipeg neighbourhood profile was used to attain a crime incidence rate per 10,000 persons for the year. A complete list of crime measures includes: arson, break and enter, firearm offensive weapon, mischief, prostitution, robbery, sexual assault, thefts motor vehicle, thefts over $5,000 and thefts under $5,000. 

### 2.5. Statistical Analysis

Census tract indicators from the community socioeconomic makeup and community disorder domains on a continuous scale were categorized into class intervals to assign quintile values used in assigning factor levels. A standard format was applied in respondents to a neighbourhood stressor quintile score from the 1996 Canada Census [41]. Quintile 1 corresponded to the census tracts with the lowest frequency of the population with the profile characteristic, and quintile 5 to the highest. Statistical analysis was performed using two subsets of children stratified from the SAGE survey: (1) those resident of birth homes at age 7 or 8; and (2) birth and non-birth home children in Winnipeg at age 7 or 8. Frequency counts per each category of individual-level measures from the SAGE survey and community stressor were calculated, to check that cell counts did not contain fewer than 5 observations. Cross tabulations between parent report of child asthma and each community stressor were explored with Pearson’s Chi-squared tests. 

A random-intercept, binomial mixed model with fixed predictor at the individual-level was fitted, as is common in ecologic models of dichotomous outcome fitting retrospective or observational data [34,42]. The grouping indicator to examine community-level variance was the City of Winnipeg neighbourhood profile in all analyses. A null model was constructed to examine the proportion of community-level variance in child asthma explained by the grouping unit, with deviations from zero suggesting differences between neighbourhood profiles. From this model, community-level and individual-level variables were added from the list of indicators that exhibited significance (*P* < 0.10) from Chi-squared tests performed in bivariate analysis. All models with the exception of the null model adjusted for the incidence of community thefts over $5,000 in neighbourhoods, respectively amongst the strata of children resident of birth homes and birth and non-birth home children in Winnipeg. Adjustment for community crime (thefts over $5,000) in addition to community socioeconomic makeup and community disorder was attempted using the measures found significant from bivariate examination under those domains. The PROC GLIMMIX function in SAS version 9.2 (SAS Institute Inc, Cary, North Carolina) was used for all multilevel analysis.

## 3. Results

Among the 1472 children living in Winnipeg neighbourhoods at age 7 or 8, 13.4% had been reported by a caregiver as having asthma (Table 1). Asthma prevalence was 12% among the 698 children that had continued to reside in their birth home. Compared to birth-home children, a higher frequency of all Winnipeg children had mothers (9.8%) or fathers (8.2%) with a history of asthma. Approximately 36% of all Winnipeg children with asthmatic mothers had asthma, whereas asthma was reported in 25% of birth-home children with asthmatic mothers. Asthma prevalence was highest among children with hay fever, and those exposed to household tobacco smoke or mold. Examining child asthma with regards to community stressors variables under the domains of socioeconomic makeup and community disorder, asthma was more prevalent among children living in low stressor or more affluent census tract quintiles. However, almost 20% of children who resided in the highest crime neighbourhoods (measured by thefts over $5,000) were reported to have asthma compared to 14.3% of children in the lowest crime neighbourhoods. Similar community stressor differences in child asthma were observed for birth home children, and they were more pronounced. 

**Figure 1:**
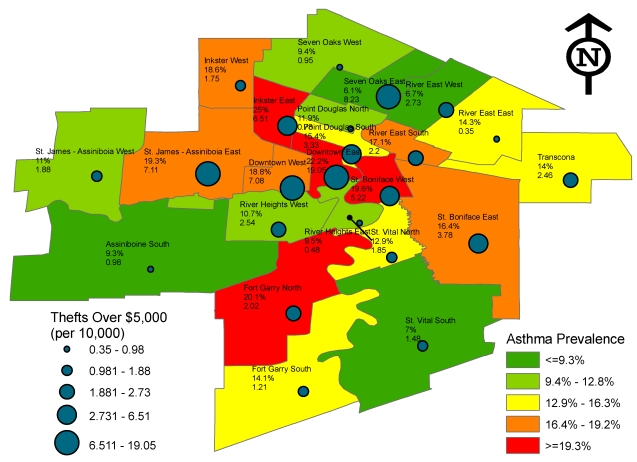
Asthma prevalence of birth and non-birth home children at age 7 or 8 and the annual incidence of thefts over $5,000 in Winnipeg by neighbourhood profile.

In terms of specific Winnipeg neighbourhoods (Figure 1), asthma prevalence was highest in Inkster East (25%), Downtown East (22.2%), Fort Garry North (20.1%) and St. Boniface West (19.6%). Many of these neighbourhoods also had the highest theft rates. High theft rates were also observed in the Downtown West, St. James-Assiniboia East and Seven Oaks East neighbourhoods. With the exception of Seven Oaks East, these neighbourhoods had child asthma prevalence rates in excess of 15%. 

**Table 1:** Characteristics of Winnipeg Schoolchildren in the Study Sample.

Variable	Category	Children of Birth Homes( *N* = 698)				Birth and Non-Birth Home Children ( *N* = 1472)				
		All Children		Children with Asthma		All Children			Children with Asthma	
		No.	%	No.	%	No.	%		No.	%
Parent Report of Child Asthma	Yes	84	12.0	NA	NA	197	13.4		NA	NA
	No	614	88.0	NA	NA	1275	86.6		NA	NA
Father has Asthma	Yes	51	7.3	16	31.4	120	8.2		31	25.8
	No	630	90.3	64	10.2	1302	88.5		151	11.6
	Not Sure	17	2.4	4	23.5	50	3.4		15	30.0
Mother has Asthma	Yes	57	8.2	14	24.6	144	9.8		52	36.1
	No	624	89.4	68	10.9	1283	87.2		140	10.9
	Not Sure	17	2.4	2	11.8	34	2.3		5	14.7
Family History of Asthma (separate variable)	Yes	162	23.2	42	25.9	390	26.5		112	28.7
	No	536	76.8	42	7.8	1082	73.5		85	7.9
Hay Fever in Child	Yes	46	6.6	24	52.2	117	7.9		59	50.4
	No	652	93.4	60	9.2	1355	92.1		138	10.2
Mold in Household 1995	Yes	5	0.7	2	40.0	208	14.1		40	19.2
	No	693	99.3	82	11.8	1264	85.9		157	12.4
Smoking in Household	Yes	148	21.2	24	16.2	357	24.3		62	17.4
	No	550	78.8	60	10.9	1115	75.7		135	12.1
Incidence of Low Income in Households	Quintile 1	52	7.4	10	19.2	135	9.2		21	15.6
	Quintile 3	173	24.8	16	9.2	325	22.1		34	10.5
Households that are Home Owner Households	Quintile 2	164	23.5	14	8.5	339	23.0		36	10.6
	Quintile 5	47	6.7	9	19.1	123	8.4		24	19.5
Income Government Transfers Comprise	Quintile 1	50	7.2	10	20.0	145	9.9		24	16.6
	Quintile 5	203	29.1	21	10.3	438	29.8		45	10.3
Aboriginal Population Composition	Quintile 1	53	7.6	10	18.9	143	9.7		22	15.4
	Quintile 4	128	18.3	10	7.8	284	19.3		29	10.2
Median 1995 Income	Quintile 1	233	33.4	26	11.2	467	31.7		55	11.8
	Quintile 5	41	5.9	10	24.4	121	8.2		21	17.4
Male Population Aged 15+ and Unemployed	Quintile 1	44	6.3	12	27.3	148	10.1		28	18.9
	Quintile 2	154	22.1	19	12.3	271	18.4		40	14.8
	Quintile 3	157	22.5	13	8.3	338	23.0		48	14.2
	Quintile 4	172	24.6	16	9.3	351	23.8		36	10.3
Population that is Male Aged 15 to 24	Quintile 1	186	26.6	16	8.6	397	27.0	42		10.6
	Quintile 3	147	21.1	23	15.6	307	20.9	55		17.9
Labour force Participation Aged 15 to 64	Quintile 1	219	31.4	22	10.0	457	31.0	50		10.9
	Quintile 4	109	15.6	20	18.3	242	16.4	42		17.4
Homes Needing Major Repairs	Quintile 2	107	15.3	22	20.6	228	15.5	39		17.1
	Quintile 5	195	27.9	22	11.3	428	29.1	51		11.9
Sexual Assaults per 10,000 Persons	2.03	56	8.0	5	8.9	122	8.3	13		10.7
	9.08	52	7.4	7	13.5	91	6.2	10		11.0
Thefts Over $5,000 per 10,000 Persons	0.35	49	7.0	5	10.2	105	7.1	15		14.3
	7.11	41	5.9	7	17.1	83	5.6	16		19.3

Abbreviation: NA, not applicable.

All community stressors were analyzed for significance in bivariate and multivariate models with regards to the dependent outcome of parent report of child asthma (Table 2). Models were adjusted for household tobacco smoke and mold, and for maternal, paternal or family asthma. The majority of community stressor variables under the *socioeconomic makeup* and *community disorder* domains were inversely associated with child asthma. Two community crime stressors, sexual assaults and thefts over $5,000 showed a statistically significant positive association with childhood asthma. Among children residing in their birth homes, neighbourhoods with thefts over $5,000 exhibited a higher risk for child asthma after adjusting for a respondent’s hay fever, father’s asthma status, and mold in house. Specifically, birth home children residing in the higher theft neighbourhood of St. James-Assiniboia East were over twice more likely (Adjusted OR, 2.10; 95% CI, 1.14–3.86) to have report of asthma by caregiver than children residing in the lower theft neighbourhood of River East East. The strength of this association was mitigated with the inclusion of birth and non-birth home children resident of Winnipeg at age 7 or 8, with children in St. James-Assiniboia East having roughly one and a half times the odds (Adjusted OR, 1.47; 95% CI, 1.00–2.16) for parent report of asthma compared to children in River East East.

Multilevel models adjusting for stressors from the *community socioeconomic makeup* and *community disorder* domains in addition to the community crime stressor thefts over $5,000 were fitted (Table 3). The null model that tested for the variation in neighbourhood asthma without adjusting for any factors showed a variation of 12% between neighbourhood profiles for children living in birth homes at age 7 or 8, and a variation of 5% for children resident of both birth and non-birth homes. Among children resident of birth homes at age 7 or 8, the community crime stressor by itself explained 58.3% of the neighbourhood variation in asthma observed [from values for neighbourhood variance: (Model I−Model II)/Model I], while among children resident of birth and non-birth homes this percentage was 60%. The community socioeconomic stressor explained approximately 58.3% of the neighbourhood variation in asthma among children resident of birth homes [from values for neighbourhood variance: (Model III − Model IV)/Model I], while it explained 40% of the neighbourhood asthma variation among children resident of both birth and non-birth homes.

**Table 2:** Likelihood of Parent Reported Asthma in Winnipeg Schoolchildren According to the Presence of Community Stressors.

Variable	Reference Category		Likelihood of Asthma at Age 7 or 8, OR (95% CI)			
			Children of Birth Homes		Birth and Non-Birth Home Children	
	Quintile or Crime Value	Quintile Proportion, Income or Profile Name	Unadjusted	Adjusted	Unadjusted	Adjusted
*Community Socioeconomic Makeup Domain*						
Incidence of Low Income in Households	Quintile 3 to Quintile 1	[0.17, 0.25) to [0.01, 0.10)		0.38 (0.15–0.95) ^a^		
Households that are Home Owner Households	Quintile 5 to Quintile 2	[0.88, 0.99) to [0.46, 0.65)	2.79 (1.07–7.30)	3.49 (1.30–9.37) ^b^	2.05 (1.13–3.72)	1.87 (1.01–3.46) ^b^
Income Government Transfers Comprise	Quintile 5 to Quintile 1	[0.22, 0.53) to [0.03, 0.09)		0.36 (0.14–0.96) ^b^		
Aboriginal Population Composition	Quintile 4 to Quintile 1	[0.06, 0.10) to [0.00 to 0.02)	0.36 (0.13–0.96)	0.32 (0.11–0.92) ^c^		
Median 1995 Income	Quintile 5 to Quintile 1	[$23,233, $34,279) to [$8,710, $15,184)	2.48 (1.02–6.05)	3.06 (1.17–8.03) ^d^		
Male Population Aged 15+ and Unemployed	Quintile 4 to Quintile 1	[0.09, 0.13) to [0.00, 0.04)	0.27 (0.11–0.66)	0.24 (0.09–0.66) ^d^	0.50 (0.28–0.88)	0.50 (0.27–0.92) ^d^
	Quintile 3 to Quintile 1	[0.07, 0.09) to [0.00, 0.04)	0.25 (0.10–0.61)	0.23 (0.08–0.63) ^d^		
	Quintile 2 to Quintile 1	[0.04, 0.07) to [0.00, 0.04)	0.37 (0.16–0.87)	0.37 (0.14–0.95) ^d^		
*Community Disorder Domain*						
Population that is Male Aged 15 to 24	Quintile 3 to Quintile 1	[0.06, 0.07) to [0.03, 0.06)		2.31 (1.11–4.80 )^e^	1.84 (1.18–2.88)	1.99 (1.24–3.17) ^e^
Labour Force Participation Aged 15 to 64	Quintile 4 to Quintile 1	[0.69, 0.75) to [0.42, 0.57)	2.14 (1.03–4.43)	2.28 (1.02–5.10) ^d^	1.70 (1.05–2.74)	
Homes Needing Major Repairs	Quintile 5 to Quintile 2	[0.13, 0.21) to [0.05, 0.07)	0.49 (0.24–0.99)	0.47 (0.23–0.98) ^e^		
*Community Crime and Violence Domain*						
Sexual Assaults per 10,000 Persons	9.08 to 2.03	St. James-Assiniboia West to River Heights West	1.21 (0.99–1.47) ^†^	1.25 (1.01–1.55) ^d^	1.12 (0.99–1.27) ^‡^	1.16 (1.02–1.32) ^d^
Thefts Over $5,000 per 10,000 Persons	7.11 to 0.35	St. James-Assiniboia East to River East East	1.85 (1.06–3.21)	2.10 (1.14–3.86) ^d^	1.42 (0.99–2.02) ^†^	1.47 (1.00–2.16) ^d^

Abbreviations: CI, confidence interval; OR, odds ratio; ^a^ Adjusted for father has asthma, mold in household 1995; ^b^ Adjusted for mother has asthma, father has asthma; ^c^ Adjusted for father has asthma, hay fever in child; ^d^ Adjusted for father has asthma, hay fever in child, mold in household 1995; ^e^ Adjusted for family history of asthma, smoking in household. † *P* < 0.06; ‡ *P* < 0.07.

**Table 3:** Likelihood of Asthma in Winnipeg Schoolchildren According to Community Crime after Controlling for Other Community Stressor Domains and Potential Confounders.

Children of Birth Homes at Age 7 or 8						
Variable	Reference Category	Model I: null model	OR (95% CI)			
			Model II: addition of crime	Model III: addition of participant data	Model IV: addition of community social makeup to Model III	Model V: addition of community disorder to Model III
Homes Needing Major Repairs	Quintile 5 to Quintile 2					0.67 (0.30–1.50)
Male Population Aged 15+ and Unemployed	Quintile 4 to Quintile 1				0.32 (0.12–0.91)	
	Quintile 3 to Quintile 1				0.32 (0.11–0.92)	
	Quintile 2 to Quintile 1					
Father has Asthma	Yes to No			4.14 (2.06–8.33)	4.22 (2.04–8.72)	3.93 (1.90–8.11)
	Not Sure to No					
Hay Fever in Child	Yes to No			11.36 (5.85–21.74)	10.71 (5.37–21.35)	11.49 (5.75–22.73)
Mold in Household 1995	Yes to No			7.52 (1.22–45.45)	9.43 (1.46–62.5)	6.45 (1.00–41.67)
Smoking in Household	Yes to No			1.61 (0.93–1.61) ^†^		1.65 (0.94–2.90) ^†^
Thefts Over $5,000 per 10,000 Persons	7.11 to 0.35		1.85 (1.06–3.21)	2.05 (1.11–3.81)	1.82 (0.96–3.46) ^‡^	1.94 (1.00–3.71)
Neighbourhood Variance (SE)		0.12 (0.13)	0.05 (0.11)	0.14 (0.16)	0.07 (0.15)	0.11 (0.15)
Median OR		1.13	1.05	1.15	1.07	1.12
**Birth and Non-Birth Home Children at Age 7 or 8**						
Homes Needing Major Repairs	Quintile 5 to Quintile 2					0.75 (0.44–1.29)
Male Population Aged 15+ and Unemployed	Quintile 4 to Quintile 1				0.61 (0.32–1.16)	
	Quintile 3 to Quintile 1				0.93 (0.50–1.73)	
	Quintile 2 to Quintile 1					
Father has Asthma	Yes to No			2.58 (1.60–4.18)	2.63 (1.62–4.28)	2.50 (1.54–4.07)
	Not Sure to No			3.41 (1.75–6.65)	3.62 (1.84–7.12)	3.46 (1.77–6.76)
Hay Fever in Child	Yes to No			8.70 (5.71–13.16)	8.80 (5.76–13.45)	
Mold in Household 1995	Yes to No					
Smoking in Household	Yes to No			1.45 (1.02–2.06)	1.41 (0.98–2.02) ^‡^	1.43 (1.00–2.04)
Thefts Over $5,000 per 10,000 Persons	7.11 to 0.35		1.42 (0.99–2.02) ^†^	1.46 (0.99–2.14) ^†^	1.46 (0.97–2.20) ^‡^	1.38 (0.91–2.11)
Neighbourhood Variance (SE)		0.05 (0.05)	0.02 (0.04)	0.03 (0.05)	0.01 (0.05)	0.04 (0.06)
Median OR		1.05	1.02	1.03	1.01	1.04

Abbreviations: CI, confidence interval; OR, odds ratio; † *P* < 0.06; ‡ *P* < 0.07.

Adjusting for individual risk factors and thefts over $5,000, children resident of birth homes in neighbourhood profile St. James-Assiniboia East were twice as likely (Adjusted OR, 2.05; 95% CI, 1.11–3.81) to have report of asthma compared to children in profile River East East, with community thefts over $5,000 explaining 14% of the neighbourhood variation in asthma. After including the covariate for community quintile proportion of male population aged 15+ and unemployed to this model, the proportion of neighbourhood variation in asthma explained was 41.7% [from values for neighbourhood variance: (Model I − Model IV)/Model I] and the significance of thefts over $5,000 decreased (*P* < 0.07) (Table 3). However, the community disorder stressor in combination with participant data and community crime stressor explained only 8.3% of the neighbourhood variation in asthma [from values for neighbourhood variance: (Model I − Model V)/Model I]. No socioeconomic makeup or community disorder stressor could be fitted that attributed significantly to the multilevel model adjusting for thefts over $5,000 involving birth and non-birth home children. Additionally, a multilevel model could not be fit for the community incidence of sexual assaults in neighbourhood profile that adjusted for census tract indicators from the 1996 Canada Census. 

## 4. Discussion

This observational study found that community stressors related to socioeconomic makeup among community disorder and crime domains contributed substantially to neighbourhood inequalities in parent reporting of child asthma amongst Winnipeg children. After adjusting for family history of asthma, smoking, and mold in household, children residing in high stress census tracts (characterized by *community socioeconomic makeup* and *community disorder domains*) were shown to have a lower likelihood of reported asthma by caregivers in comparison to children residing in the lowest stress quintile census tracts. This association was observed with the following community stressors: prevalence of low income households, households that are home owner households, income government transfers, Aboriginal population composition, median 1995 population, male population aged 15+ and unemployed, labour force participation aged 15 to 64, and homes needing major repairs. Although these results went against our initial hypothesis, the association between high census tract quintile SES and increased asthma prevalence has been observed by other studies [15,31]. On the other hand, children resident of high crime neighbourhood profiles had a much higher likelihood for report of asthma by caregiver in comparison to children resident of low crime neighbourhoods.

A striking differential was observed in the strength of the association between community stressors and child asthma in our sensitivity analyses of birth home children. Asthma was twice more likely, independent of household factors, among children living in the same high crime neighbourhood since birth. It was 1.5 fold more likely and of marginal statistical significance in all Winnipeg children living in high crime neighbourhoods. Additionally, it was found from the multilevel models that community thefts over $5,000 explained a larger proportion of the neighbourhood variation in asthma prevalence among children resident of birth homes at age 7 or 8, when compared to birth and non-birth home children in Winnipeg. There are at least 2 possible explanations for these differences. Firstly, community stressors may have a cumulative effect in promoting asthma development among children who have lived in the same neighbourhood from the time of birth [9]. Second, parents of asthmatic children may have moved, potentially diluting the effect of the neighbourhood. The prevalence of asthmatic children with an asthmatic mother was higher (36.1%) among families that had relocated homes since time of child’s birth, in comparison to asthmatic children with asthmatic mothers who were resident of birth homes (24.6%). 

Small to large proportions of the variance in child asthma at the neighbourhood profile level were explained by *community socioeconomic makeup* or *community disorder* stressors at the census tract level, in comparison to the null model fitting no stressors or participant data. When compared to the model adjusting for community crime, it is apparent that thefts over $5,000 are the driver of the variation observed in neighbourhood child asthma prevalence. Interestingly, after the addition of participant data for children resident of birth homes at age 7 or 8 (Table 2), the variation in asthma by neighbourhood profile actually increased, and was reduced by 50% when adjusting for community socioeconomic makeup in census tract quintiles asthma [from values for neighbourhood variance: (Model III − Model IV)/Model IV]. A possible explanation for this is that even though the City of Winnipeg neighbourhood profiles may be homogeneous, the census tracts nested within neighbourhood profiles are heterogeneous with respect to SES. While central area neighbourhoods in the downtown region of Winnipeg were relatively homogeneous, large variations in community socioeconomic makeup stressors was observed amongst neighbourhood profiles in the outlaying parts of the city. Due to Winnipeg’s history as an amalgamated municipality of 13 smaller communities [43], a homogeneous downtown core with wide differences in SES among inner-city and outlying neighbourhoods seems plausible, given a physical environment consisting of historic and infill housing with a variety of build times [44]. 

Our study is the first to investigate the association between community crime and violence, and asthma prevalence amongst children in Canada, and is among a few that have been published worldwide [23,34,45,46,47]. The findings we obtained are suggestive of a pathway that exists between chronic psychosocial stress from community victimization, and the prevalence of asthma in children. From the literature, chronic fluctuations in the level of corticosteroids induced by social stressors can cause allostatic load that makes it easier for stimuli to elicit restriction of airways [48]. While stress hormones are part of the immune system response to rid the body of infection, high concentrations of glucocorticoids are responsible for immunosuppression in tissues that may be stress-induced [49]. Prolonged play of these mechanisms from chronic exposure to stressors in a neighbourhood also increases the susceptibility for allergens and other stimuli to trigger an asthmatic attack in children. However, our analysis stopped short of linking individual-level maternal depression scores within households to caregiver report of child asthma, and stressors at the ecologic level in Winnipeg. Examining this relationship is clearly the next step.

## 5. Limitations

Several methodological caveats of this research should be acknowledged. First, child asthma was self-reported by caregivers, with the potential of misclassifying this outcome. Secondly, the study was ecological in nature and suffers from the fallacy that not all children residing in a specific census tract or neighbourhood exhibit the same characteristics in terms of stressors which define the neighbourhood. Thirdly, a response rate of 28.6% in the SAGE survey is modest, and it is unknown if participants differ from the population of non-responders in making inferences to the sampling frame of cohort children. With respect to the first caveat, a sensitivity and specificity analysis was performed on a subgroup of 102 SAGE survey children resident of birth homes at age 9. Comparing caregiver report of asthma from the SAGE survey in this subgroup of children to pediatric allergist diagnosis of asthma (which is used as the gold standard), a high sensitivity of 84.1% and a high specificity of 88.4% were obtained [50]. 

## 6. Conclusions

Children resident of higher stress census tract quintiles under the domains of community *socioeconomic makeup* and *community disorder* had a lower likelihood of asthma being reported at age 7 or 8 by a caregiver. However, children residing in high crime neighbourhoods were much more likely to have parent-reported asthma after adjusting for other community stressor domains and individual-level risk factors. The latter findings highlight the negative influence of neighbourhood stress in early childhood on asthma, either by making symptoms worse or leading to its development subsequent to inducing stress in the mother during pregnancy and postnatally. Further investigation is warranted to examine the association between community crime and asthma prevalence of children in neighbourhoods, and the pathway this relates to maternal stress and depression. In the interim, public health nurses, health care providers and teachers should be made aware that growing up in a stressful environment affects physical, as well as mental health. They also should be alerted to the fact that children who witness or experience community violence are also at heightened risk of child maltreatment [9]. From a city planning and protection perspective, creating and maintaining safe neighbourhoods by for example, reducing crime and improving social networks, benefits the health of all of its residents. 
